# Author Correction: A high-performance capillary-fed electrolysis cell promises more cost-competitive renewable hydrogen

**DOI:** 10.1038/s41467-024-52303-8

**Published:** 2024-09-11

**Authors:** Aaron Hodges, Anh Linh Hoang, George Tsekouras, Klaudia Wagner, Chong-Yong Lee, Gerhard F. Swiegers, Gordon G. Wallace

**Affiliations:** 1 Intelligent Polymer Research Institute, University of Wollongong, Wollongong, NSW 2522 Australia; 2 Australian Research Council Centre of Excellence for Electromaterials Research, University of Wollongong, Wollongong, NSW 2522 Australia

Correction to: *Nature Communications* 10.1038/s41467-022-28953-x, published online 15 March 2022

The original manuscript contains a calculation error in ref. 2. This has been corrected in the PDF and HTML versions of the Article. A limitation statement of the calculation in ref. 2 is also added.

Original ref. 2:

Green Hydrogen Cost Reduction: Scaling up Electrolysers to Meet the 1.5 °C Climate Goal. (International Renewable Energy Agency, Abu Dhabi, 2020). OPEX is, by far, the major component of the LCOH for the following reason: Fig. ES1 in this reference provides an average electrolyser CAPEX (today) of USD$770 per 1 kW capacity. Over 10 years of continuous operation the electricity consumed by that 1 kW capacity, which will comprise most of the OPEX, will be: 24 × 365 × 10 = 1,752,000 kWh. At the average electricity price of USD$53/MWh, this gives a total OPEX of at least USD$91,104, which is 118-times larger than the CAPEX. The dominance of the OPEX over the CAPEX in the LCOH is projected to increase. Using the best future CAPEX of USD$130/kW and electricity price of USD$20/MWh over 10 years of continuous operation: the OPEX of USD$35,040 will be 270-times larger than the CAPEX. The IRENA 2050 cell energy consumption target is given in Table 6 (Stack electrical efficiency).

Updated ref. 2:

Green Hydrogen Cost Reduction: Scaling up Electrolysers to Meet the 1.5 °C Climate Goal. (International Renewable Energy Agency, Abu Dhabi, 2020). OPEX is, by far, the major component of the LCOH for the following reason: Fig. ES1 in this reference provides an average electrolyser CAPEX (today) of USD$770 per 1 kW capacity. Over 10 years of continuous operation the electricity consumed by that 1 kW capacity, which will comprise most of the OPEX, will be: 24 × 365 × 10 = 87,600 kWh. At the average electricity price of USD$53/MWh, this gives a total OPEX of at least USD$4,642.80, which is 6-times larger than the CAPEX. The dominance of the OPEX over the CAPEX in the LCOH is projected to increase. Using the best future CAPEX of USD$130/kW and electricity price of USD$20/MWh over 10 years of continuous operation: the OPEX of USD$1,752 will be 13.5-times larger than the CAPEX. The IRENA 2050 cell energy consumption target is given in Table 6 (Stack electrical efficiency). It should be noted that this calculation provides only a general order of magnitude comparison of OPEX and CAPEX. It ignores financing costs and discounting of payments that occur over time.

