# Supplementary material for: A high-performance capillary-fed electrolysis cell promises more cost-competitive renewable hydrogen
Source: Nat Commun. 2022 Mar 15;13:1304. doi: 10.1038/s41467-022-28953-x (PMC8924184; doi:10.1038/s41467-022-28953-x)
Supplement: Supplementary file 1 — Supplementary Information [file 41467_2022_28953_MOESM1_ESM.pdf]

Supplementary Information

A high-performance *capillary-fed electrolysis* cell promises more cost-competitive renewable hydrogen

Aaron Hodges[1], Anh Linh Hoang[1], George Tsekouras[1], Klaudia Wagner[1,2], Chong-Yong Lee[1,2], Gerhard F. Swiegers[1,2,*] & Gordon G. Wallace[1,2]

Supplementary Fig. 1

Example illustrating the effect of an increased energy efficiency on the levelized cost of hydrogen (LCOH).

Definition of energy efficiency:

The energy efficiencies reported in this work are for the electrolysis cell only.

The energy efficiency of an electrolysis cell is defined as the net energy present in the hydrogen produced by the cell divided by the net energy consumed by the cell to produce it, expressed as a percentage.

The net energy present in hydrogen is its *higher heating value* (HHV), which is 39.4 kWh/ kg of hydrogen.

An electrolysis cell operating at a cell potential equal to the thermoneutral voltage of water electrolysis (1.48 V at room temperature, 1.47 V at 80-85 ^{o}C) displays 100% energy efficiency.

The effect of energy efficiency on LCOH:

Illustrative example showing the impact on the levelised cost of hydrogen (LCOH) resulting from a 20% increase in the energy efficiency of an electrolyser, from 75% to 95% (HHV). Common assumptions: Electricity cost = $25/MWh, electrolyser CAPEX = $400/kW, electrolyser capacity factor = 65%, electrolyser lifetime = 20 years, weighted average cost of capital (WACC) = 6.5%, operations and maintenance (O&M) cost = 2% of electrolyser CAPEX p.a., cost of water = $0.002/kg.

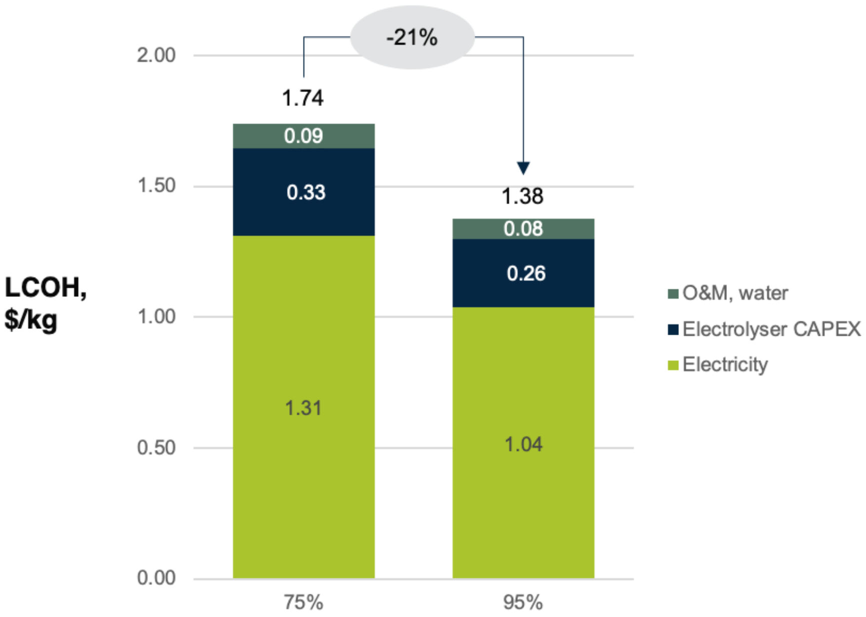

Supplementary Fig. 2

Schematic depiction of a bipolar stack of capillary-fed electrolysis (CFE) cells showing the reactant- (H_2O), product- (H_2 and O_2), and electron-flows.

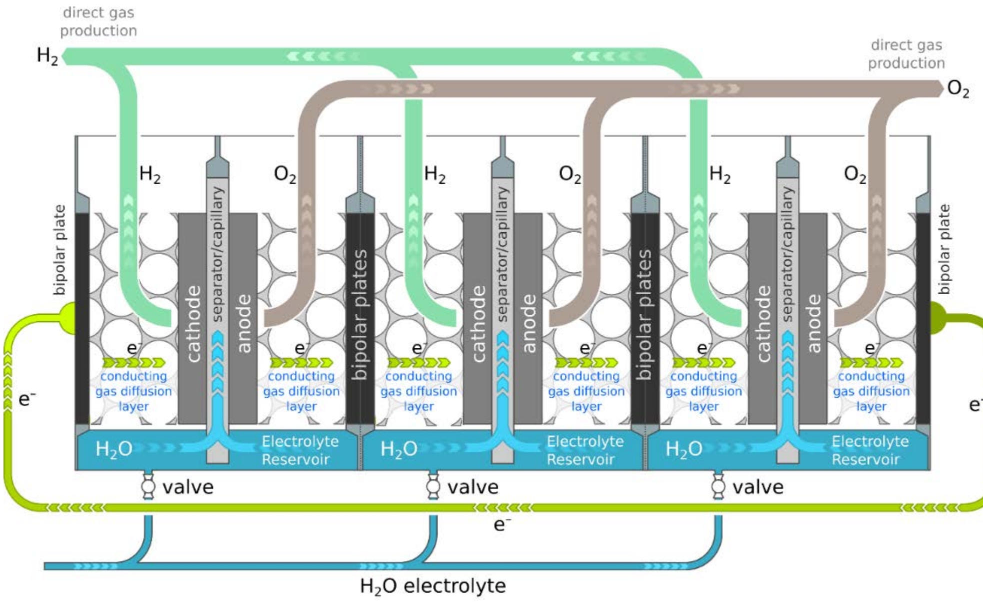

Supplementary Fig. 3

Schematic comparison of:

(a) *a conventional, bubbled electrolysis bipolar cell stack and balance of plant (Reproduced and adapted from ref. 1 with permission from the Royal Society of Chemistry), and*

(b) *a capillary-fed electrolysis (CFE) bipolar cell stack and balance of plant.*

Legend
- M = Inter-electrode membrane/ionomer
- B = Bipolar electrode
- V = De-ionized water valve

(a) Conventional, bubbled electrolysis cell stack and balance of plant [1]

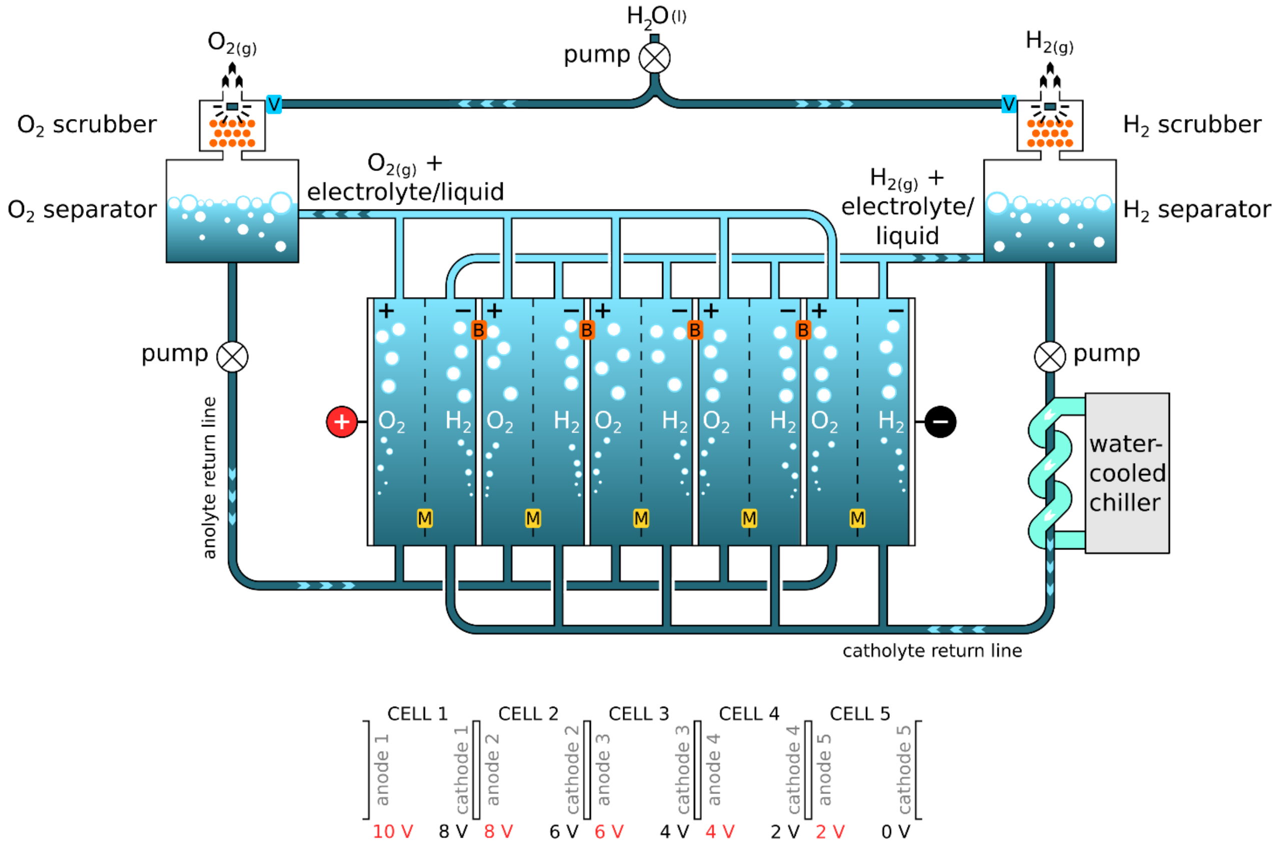

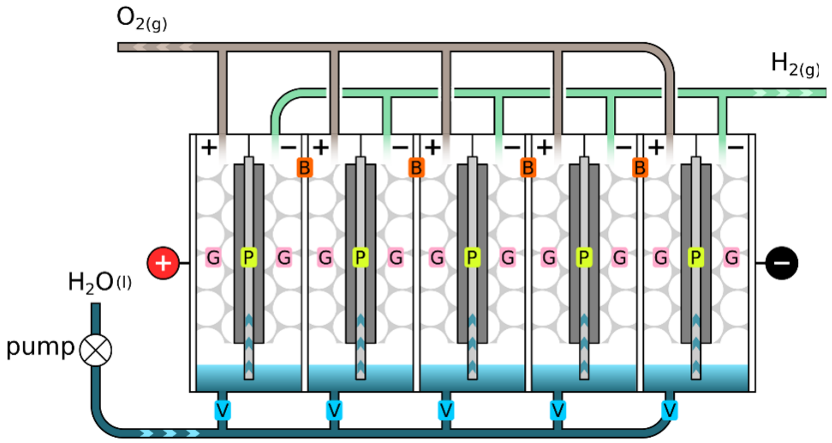

(b) Capillary-fed electrolysis cell stack and balance of plant

Legend
- P = PES membrane/electrode assembly
- B = Bipolar plate
- V = De-ionized water valve
- G = Conducting gas diffusion layer

Supplementary Note 1

Operation of a scaled-up capillary-fed cell in a cell stack of the type used in commercial electrolysers.

Comparison of the heat removal requirements of alkaline, PEM, and capillary-fed electrolysis (CFE) cells and cell stacks.

The tables below compare the operating parameters, at a constant 85 ^oC (ambient temperature 20 ^oC), of conventional commercial alkaline and PEM cells and stacks, with capillary-fed cells and stacks, having the same output of hydrogen and oxygen. As can be seen at a fixed current density of 0.5 A cm^{-2}, all of the cells and stacks are self-heating. Once they achieve an operating temperature of 85 ^oC, they produce different quantities of waste heat that must be removed by cooling.

In broad terms, cooling capacities exceeding ~7.5-10 kW require water-cooled chillers. Below that, air-cooling may potentially be used. At very low cooling capacities, radiative self-cooling of the stack may be used. Radiative self-cooling would typically involve the cell or stack directly radiating the excess heat via, for example, external cooling fins. Air-cooling could be achieved by circulating a heat transfer fluid from around the stack to a fan-cooled heat exchanger.

As can be seen in Supplementary Table 2, a capillary-fed cell stack of 150.6 kW would require cooling of only 1.1 kW, which is well within the range for air-cooling or, potentially even, radiative self-cooling.

Note: The data in Supplementary Tables 1 and 2 was calculated from the current and voltage data in Fig. 3c, using the excel sheet entitled "Excel Calculator: Heat Output of Water Electrolysis Cell or Cell Stack", which has been provided as Supplementary Data 1.

Supplementary Table 1: *Comparison of operating parameters of individual cells at 85 ^oC (ambient temperature 20 ^oC), where each individual cell has anodes/cathodes of 400 cm^2 area, a volume of 500 cm^3 and produces 0.18 kg hydrogen/day and 1.43 kg oxygen/day.*

Cell Type	Fixed current density (A cm^{-2})	Individual Cell				
		Anode/Cathode Geometric Area (cm^2)	Current (A)	Voltage (V) [a]	Total Power (W)	Net Power Radiated as Heat [b] (W)
Commercial alkaline cell	0.5	400	200	1.763	352.6	53.5
Commercial PEM cell	0.5	400	200	1.594	318.8	19.7
Capillary-fed electrolysis cell	0.5	400	200	1.506	301.2	2.1

[a] Data drawn from Fig. 3c and taken as being at 85 ^oC. [b] Takes into account the heat consumed in heating up the required replenishment water from 20 ^oC to 85 ^oC (0.12 kWh/day; where the specific heat of water is 4.2 kJ/kg K).

Supplementary Table 2: *Comparison of operating parameters of stacks of 500 of the individual cells in Supplementary Table 1 at 85 ^oC (ambient temperature 20 ^oC), where each stack produces 89.52 kg hydrogen/day and 716.00 kg oxygen/day.*

Cell Stack Type	Fixed current density (A cm^{-2})	Cell Stack				
		Number of Cells	Current (A)	Voltage (V) [a]	Total Power (kW)	Cooling Capacity Required [b] (kW)
Commercial alkaline cell stack	0.5	500	200	882	176.3	26.8
Commercial PEM cell stack	0.5	500	200	797	159.4	9.9
Capillary-fed electrolysis cell stack	0.5	500	200	753	150.6	1.1

[a] Data drawn from Fig. 3c and taken as being at 85 ^oC. [b] Takes into account the heat consumed in heating up the required replenishment water from 20 ^oC to 85 ^oC (61.09 kWh/day; where the specific heat of water is 4.2 kJ/kg K).

Supplementary Note 2

Estimation of the electrolyte volume of a capillary-fed electrolysis (CFE) cell stack.

The table below provides an estimation of the total volume of liquid electrolyte in a capillary-fed cell stack. The reservoir volume is assumed to comprise 15% of the cell volume. On that basis, the stack will contain ~400 L of electrolyte per 1 MW capacity. To that must be added the volume of water in the de-ionized water dispensing system, which may be estimated to be ~100 L /MW, giving a total volume of water of ~500 L/MW.

Supplementary Table 3: *Estimation of the electrolyte volume contained within a capillary-fed electrolysis (CFE) cell stack.*

Cell and Stack Type	Individual Cell			Cell Stack			
	Electrode Area (cm^2)	Cell Thickness (cm)	Cell Reservoir Volume (15% of cell volume) (cm^3)	Number of cells [a]	Net Volume of Liquid (L)	Stack Power [a] (kW)	Net Volume of Liquid per 1 MW (L)
Capillary-fed electrolysis cell	400	2	120	500	60	150.6	400

[a] Drawn from Supplementary Table 2.

Supplementary Fig. 4

(a) *Schematic depiction of the apparatus used to carry out the flow rate experiments in Fig. 2a.*

(b) *An example of data collected for a single height of separator.*

(c) *Schematic depiction of the flow regimes in the data in (b) above.*

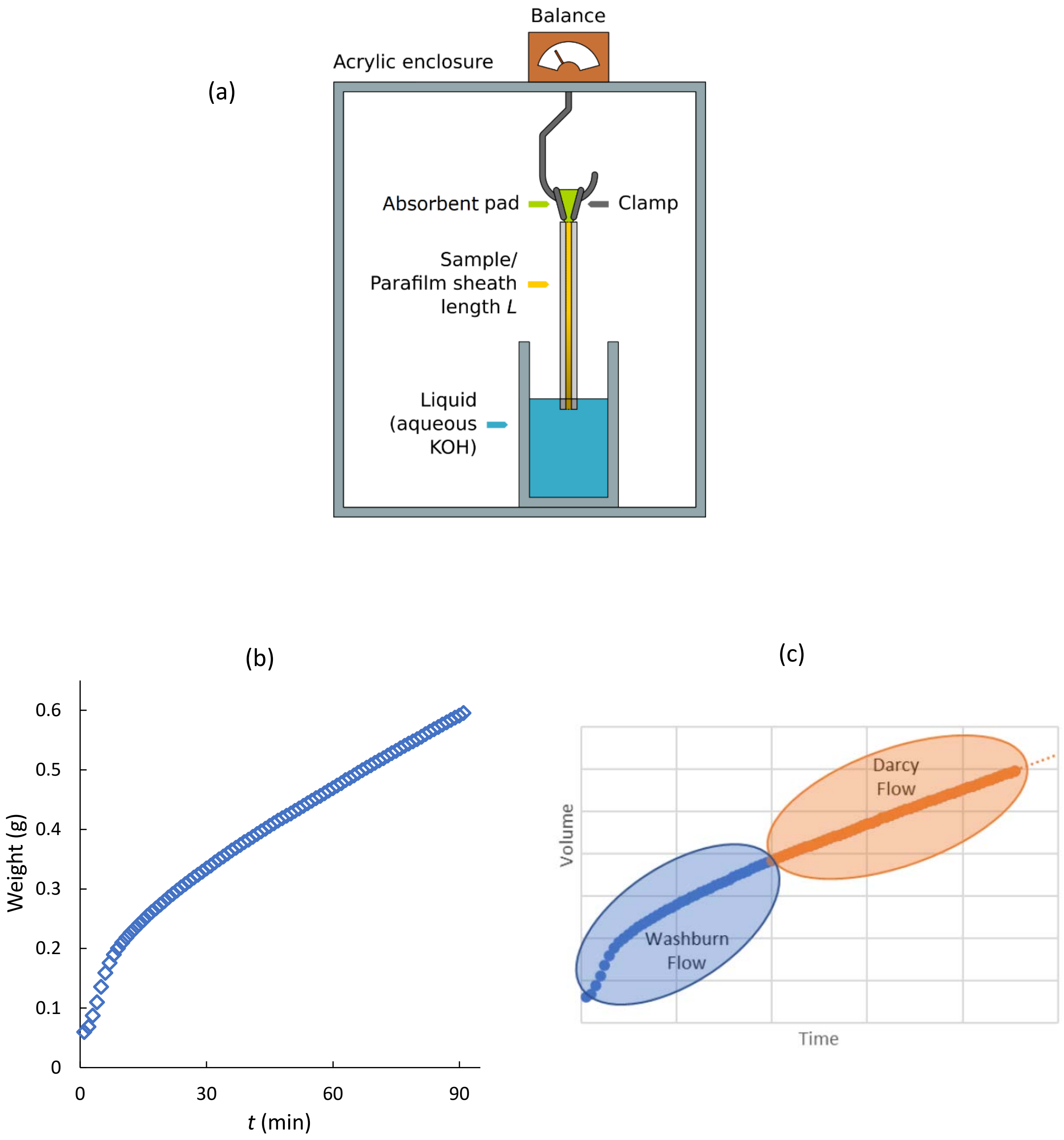

Supplementary Fig. 5

Schematic representation of the conductivity probe used to carry out the ionic resistance measurements (Reproduced without changes, from reference 2 Copyright Elsevier, DOI: /10.1016/j.jpowsour.2015.04.066, with permission under a license detailed at http://creativecommons.org/licenses/by/4.0.).

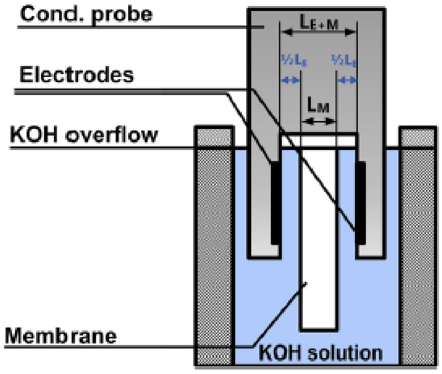

The ionic resistance of the polyether sulfone separator was determined using a method described previously[2] and the setup shown above. The conductivity of the KOH electrolyte was measured with and without an electrolyte-filled separator present using a 4-point conductivity probe (Mettler Toledo Sevencompact with Inlab ISM-731 Probe).

$$R_{\mathrm{S}} = R_{\mathrm{S+E}} - R_{\mathrm{E}} \qquad (6)$$

Equation (6) describes the separator resistance (R_{S}) as the difference between the combined resistance of the separator and electrolyte ($R_{\mathrm{S+E}}$) and the electrolyte resistance (R_{E}), as measured between the electrodes of the conductivity meter. It may be rewritten to give:

$$\rho_{\mathrm{S}} = \frac{\left(\frac{1}{\kappa_{\mathrm{E+S}}} - \frac{1}{\kappa_{\mathrm{E}}}\right) L_{\mathrm{E+S}}}{L_{\mathrm{S}}} + \frac{1}{\kappa_{\mathrm{E}}} \qquad (7)$$

Where ρ_{S} is the resistivity of the separator (including the electrolyte within), κ is conductivity, and L is as shown above.

Data obtained:

Separator	Electrolyte	Specific resistance of electrolyte [3] (Ω cm)		Thickness (µm)	Porosity (%)	Ionic Resistance (mΩ cm²)	
		25 °C	80 °C			25 °C	80 °C
Zirfon PERL UTP 500	30 wt% KOH	1.61	0.72	500 [4]	55 [4]	290 [4]	130 [e]
Polyether sulfone (8 µm)	27 wt% KOH	1.60	0.74	140	80 [5]	46 [6]	22 [7]

Supplementary Fig. 6

Illustration of the capillary-fed cell used in this work.

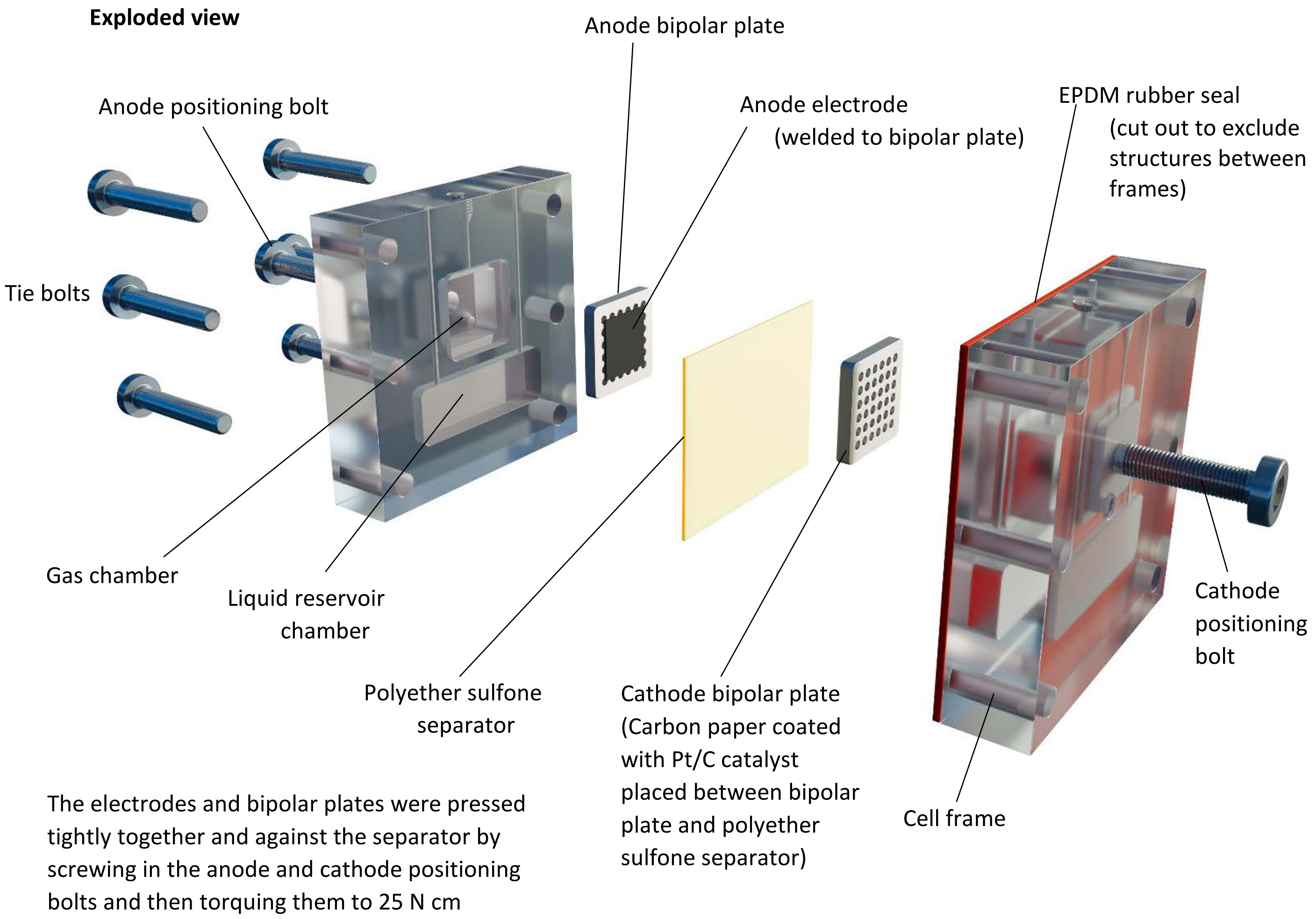

Assembled view

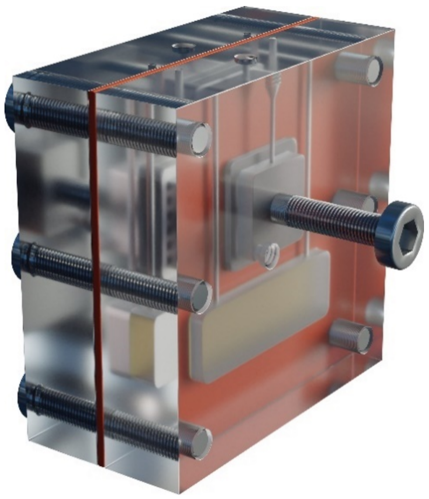

Supplementary Fig. 7

Anode preparation.

A fine Ni mesh (200 LPI) was laser cut into the shape shown below. After cutting, the Ni mesh was cleaned by ultrasonication in isopropyl alcohol for 10 min and dried, then pickled in 5 M HCl for 10 min, rinsed with deionised water, and dried. The ‘stalk’ and ‘welding locations’ were then covered with electrocoating tape, as depicted in (a) below. The sample was thereafter placed in the electrocoating solution described in the Methods section and coated with catalyst. After rinsing and drying, the electrocoating tape was removed, leaving the electrode with exposed stalk and welding locations. Image (b) shows the resulting electrode without PTFE included in the electrocoating solution. Image (c) shows the resulting electrode with PTFE included in the electrocoating solution. Electrodes prepared in this way were spot welded to their bipolar plates at the welding locations shown. Welding could be performed without loss of catalyst. The stalk was then removed, leaving the electrode affixed to its bipolar plate. All electrodes had a 1 cm^2 geometric area of catalyst. The current densities in this work are relative to the geometric area of the electrode that is covered with electrocatalyst.

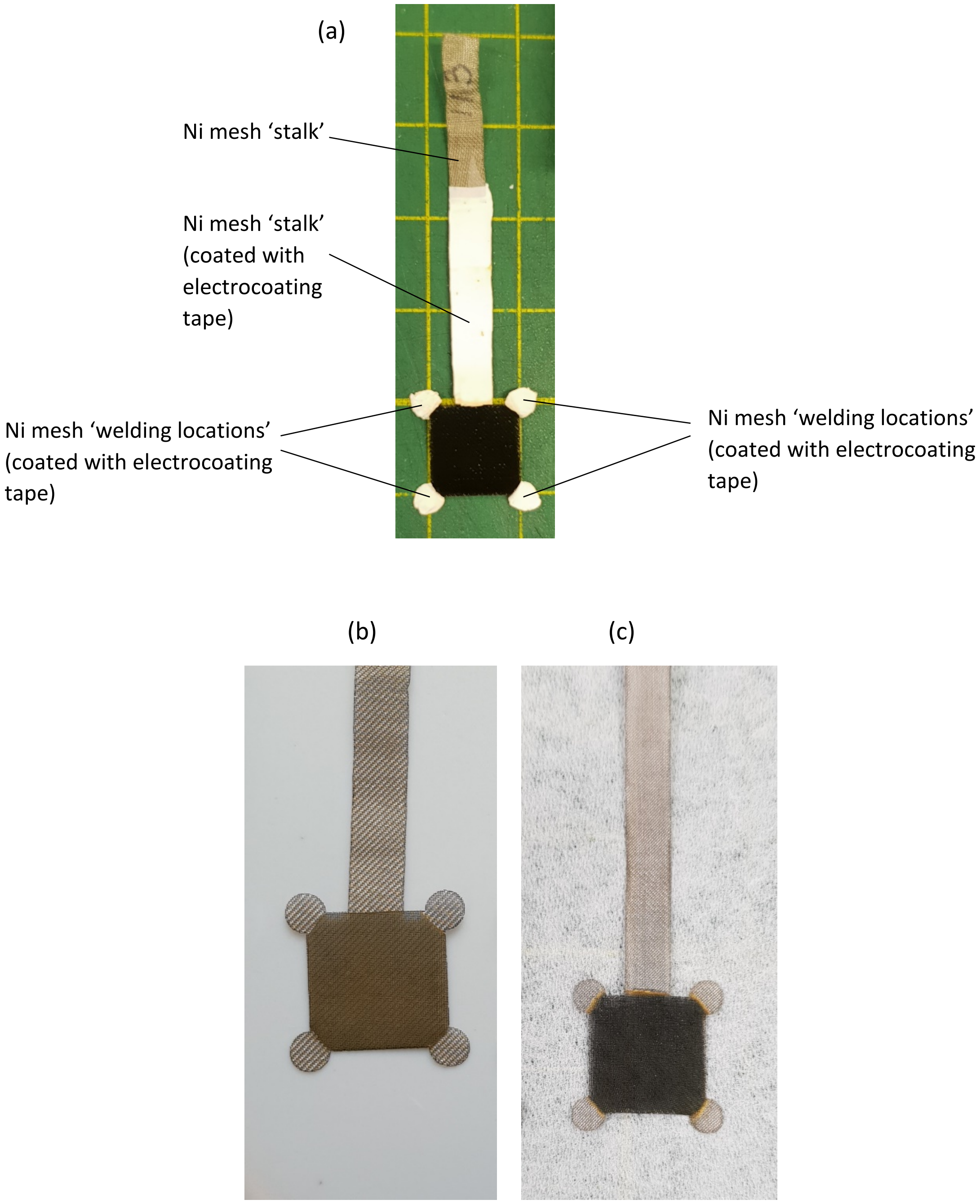

Supplementary Note 3

The structure of the cathode. Bubble-free operation by the cathode.

The cathode comprised a carbon paper gas diffusion layer (GDL) having two sub-layers:

(a) a microporous layer of carbon and PTFE binder at its front face, and

(b) a macroporous layer of PTFE-coated carbon fibres at its back face.

A thin film of Pt/C catalyst and Nafion binder was deposited on the microporous front face.

Technical data for the carbon paper GDL is provided below in Supplementary Table 4.

The graphs in Fig. 4c were determined for the capillary-fed cell itself, including the anode and cathode electrodes. Thus, both the anode and cathode were largely bubble-free up to and including 0.2 A cm^{-2}, and substantially bubble-free above that to 1 A cm^{-2}. Accordingly, the cathode exhibited, in at least some significant measure, bubble-free performance.

During operation, dissolved hydrogen may have migrated from the (wetted) catalyst layer, where it was formed, through the micro-porous front face of the GDL into the (unwetted) macroporous layer at the back. Such a movement may, conceivably, have been facilitated by the low surface energy and aerophilic nature of the PTFE in the microporous and macroporous sub-layers. This would make the process of gas removal (without bubble formation) from the cathode similar in principle to that at the anode when PTFE was incorporated.

That is, it is potentially noteworthy that the same elements for spontaneous gas migration along aerophilic PTFE surfaces, across the gas-liquid interface, were present in both the cathode and the anode, each of which displayed bubble-free operation.

Supplementary Table 4

Technical data for the carbon paper GDL used as the cathode substrate, as provided by the supplier.

**Sigracet 22BB**	
Material Type	Carbon Fibre Paper
Thickness	215 μm ± 20 μm
Areal Weight	70 ± 15 g m^{-2}
Electrical Resistivity	< 10 mΩ cm^2
Thermal Conductivity	0.30 W (m^{-1}) (K^{-1})
Bending Stiffness (MD/TD)	1.5 / 0.9 N mm
Gas Permeability	1.2 Gurley sec
Compressibility	20% (@ 5psi, 1 MPa)
Water Contact Angle (MPL side)	> 130°
Roughness (MPL side)	7.2 μm
Tensile Strength	6.9 / 4.6 MPa (MD/TD)
PTFE Treatment	5%
Microporous Layer (MPL)	Yes, on one side (the catalyst side)

Supplementary Fig. 8

Oxygen depolarisation of the capillary-fed electrolysis cell

Prior to, and between periods of operation, the gas chambers of the capillary-fed electrolysis cell were flushed with nitrogen. This was needed to avoid the presence of air-oxygen in, particularly, the cathode gas chamber. In the presence of carbon at the cathode, oxygen gas may be transformed into peroxide in a competing reaction that is ~0.7 V more favourable than the hydrogen evolution reaction (HER).[8]

Peroxide reaction under alkaline conditions: ('Oxygen depolarisation')

$$O_2(g) + 2H_2O(l) + 2e- \rightarrow H_2O_2(aq) + 2OH^-(aq) \qquad E^0_{RHE}\ 0.695\ V$$

HER under alkaline conditions:

$$2H_2O(l) + 2e^- \rightarrow H_2(g) + 2OH^-(aq) \qquad E^0_{RHE}\ 0.000\ V$$

To assess the effect of air-oxygen in the gas chambers, tests were done on the capillary-fed cell, equipped with electrodes containing catalyst, with the gas chambers filled with air. A Hg/HgO reference electrode had been sealed into the reservoir of the test cell.

The test involved measuring the cathode voltage as the current was increased in steps of, initially, 5 mA cm^{-2}, then 10 mA cm^{-2}, then 100 mA cm^{-2} and so on. The results are shown below.

As can be seen, with air-oxygen in the gas chambers, the cathode initially displayed a positive voltage vs RHE, indicating oxygen depolarisation of the type described above. As the current was stepped up, the cathode voltage declined linearly as would be expected for a competing reaction. At 30 mA cm^{-2} however, the cathode voltage vs RHE became negative, and the slope of the curve changed, indicating that the cell switched to a new half reaction, namely the HER.

It could be concluded that even with the gas chambers initially filled with air, oxygen depolarisation of the cathode persists only up to 30 mA/cm^2, whereafter hydrogen generation via the HER overwhelms it.

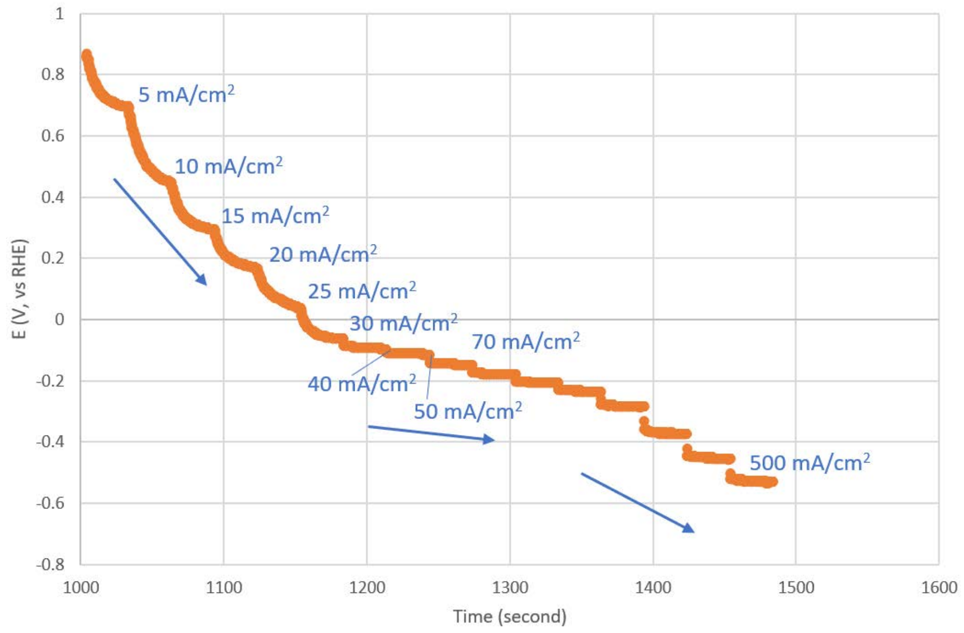

Supplementary Fig. 9

Using voltage fluctuations during bubble formation as a proxy for detecting gas bubbles

To objectively measure the incidence of gas bubble formation on the electrodes, we previously developed an electrochemical signal drift technique as a sensitive probe of bubble formation[9]. Inspired by this approach, chrono-potentiometric testing was carried out at room temperature, with each cell in Fig 3b being stepped through a series of fixed current densities. Each current density was applied to the cell for 20 s and the voltage data collected. The last 10 s at each current density was thereafter analysed and the standard deviation in the voltage measured. Representative results for the cell types in Fig 3b are shown below.

As discussed in the text, the electrodes of the capillary-fed cell with PTFE at the anode, were largely free of electrochemically detectable gas bubbles up to ~0.2 A cm^{-2}. At higher current densities, up to 1 A cm^{-2}, the performance of the capillary-fed cell indicated substantially bubble-free operation, with standard deviations similar to those of the bubbled control cells at very much lower current densities. For example, at 1 A cm^{-2}, the capillary-fed cell with PTFE at the anode displayed a standard deviation comparable to the bubbled control cells at ~0.09 A cm^{-2}, suggesting that less than 10% of the 1 A cm^{-2} current went into gas production involving bubble formation.

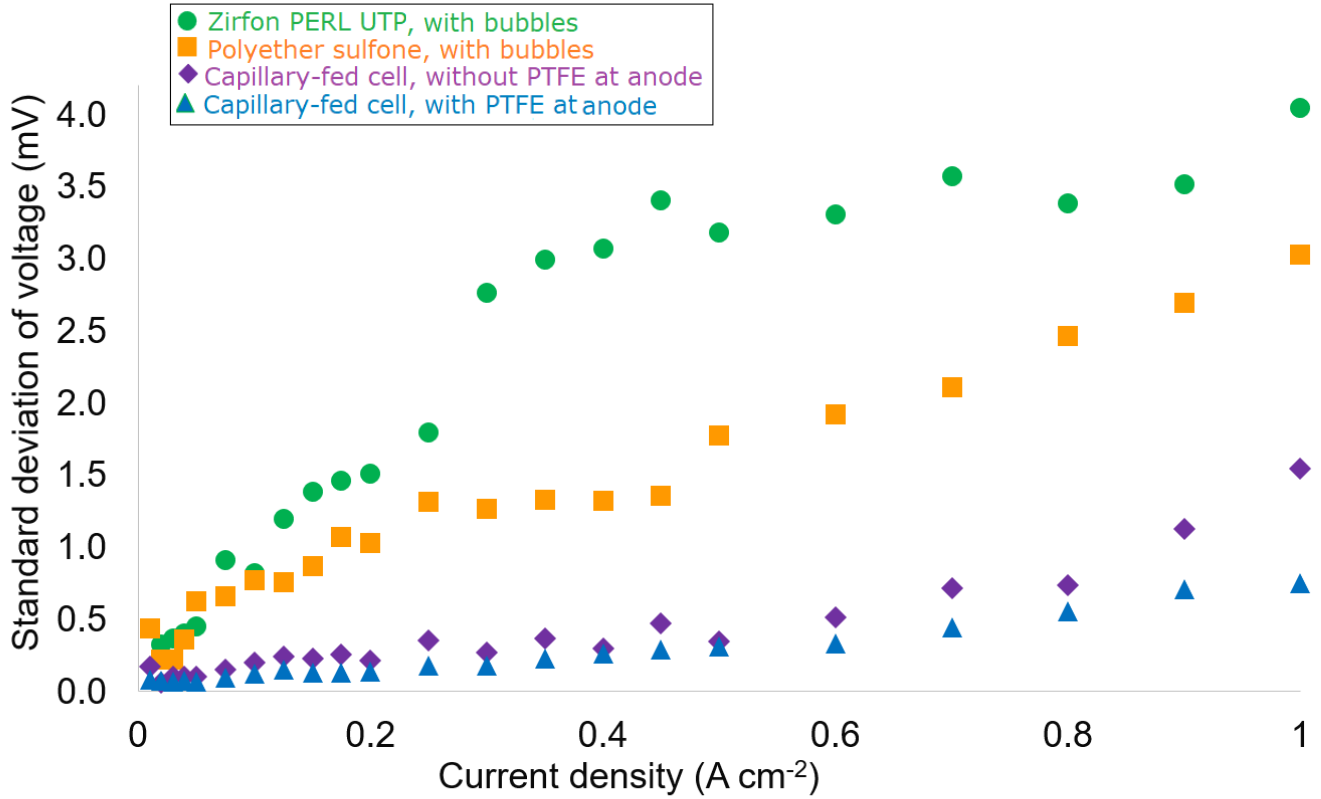

Supplementary Fig. 10

Long-term tests of the capillary-fed electrolysis cell

To undertake long-term tests of the capillary-fed cell in the absence of an automated water replenishment system, with only manual addition of make-up water, a larger reservoir was needed than the one in the test cell in Supplementary Fig. 6. This was necessary to counteract the effect of water consumption and evaporation on the KOH molarity of the electrolyte in the reservoir. Because the conductivity of the polyether sulfone separator is strongly dependent on the molarity of the KOH, changes in this respect due to water consumption and evaporation would alter cell performance. This challenge was overcome by cutting off the bottom of the cell in Supplementary Fig. 6, to remove the reservoir cavity and allow the polyether sulfone separator to extend out the bottom of the cell. The resulting cell was then placed on a stand within a 600 mL beaker, atop a body of 27 wt% KOH that had a constant stream of argon bubbling through it. The polyether sulfone separator was dipped into the 27 wt% KOH electrolyte below the cell and the cell was operated with periodic, manual additions of make-up water to the reservoir. Using this approach, the molarity of the reservoir could be reliably maintained to 27-31 wt% KOH, which has a relatively narrow range of ionic conductivity[10].

At 80 °C and a fixed cell voltage of 1.47 V: the cell needed manual replenishing about every 1½ hours, as shown below. As the cell could not be replenished during overnight operation, it was typically run during the working day, and this was repeated on the following days:

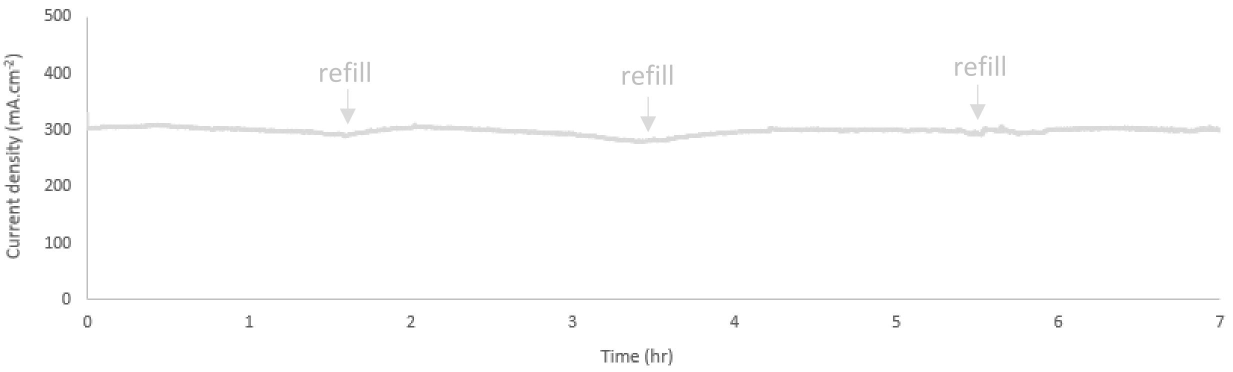

At room temperature and a fixed current of 0.4 A cm^{-2}: the cell needed manual replenishment about once per day. This allowed for monitoring of the cell performance over 30 days of continuous operation:

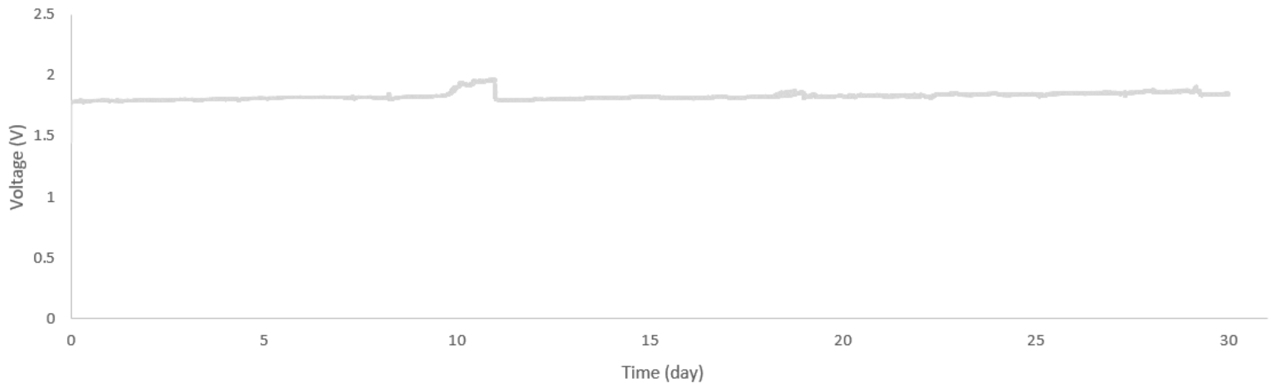

Notes:

(1) This test employed an earlier, not fully optimised version of the anode catalyst incorporating PTFE;

(2) The increase in voltage over time was 76 µV/h, which compares well with other reported cells[11,12]. The increase may have been due to a relaxation over time of the pressure with which the anode and cathode were pressed tight against the separator. While Fe deposition occurred, as in many other alkaline cells it did not interfere with catalytic function and likely only minimized Ni corrosion[13].

(3) The discontinuity in the above data at day 10-11 occurred on a long weekend when the cell could not be manually replenished. Following the addition of make-up water, the cell resumed normal operation.

Supplementary Fig. 11

GEIS as a means of elucidating the elements responsible for the high performance of the capillary-fed cell

Galvanostatic electrochemical impedance spectroscopy (GEIS) is a potentially powerful technique to determine the resistances within an electrochemical cell. It could be readily used to characterise the capillary-fed cell under active water electrolysis conditions. The relative absence of gas bubbles in the capillary-fed cell undoubtedly helped in this respect.

To establish its utility, measurements were carried out, such as the one shown below, which was performed at a current density of 0.35 A cm^{-2} at 80 °C (Conditions: 0.350 A cm^{-2} DC bias, 0.050 A cm^{-2} AC perturbation, between 100 kHz and 100 mHz).

As can be seen, the data crosses zero on the y-axis at ~0.04 Ω cm^2, i.e. 40 mΩ cm^2. This crossing point indicates the 'series resistance' of the cell, which includes all of the resistances between the electrodes.

As noted in the table at the bottom of Supplementary Fig. 5, the resistance of the KOH-imbued PES separator at 80 °C is 22 mΩ cm^2. Accordingly, most but not all of the inter-electrode resistance was attributable to the separator. The origin of the remaining resistance of 40-22 = 18 mΩ cm^2 was not clear. It could have been due to stray bubble formation, but this could not be unequivocally discerned.

Accordingly, while useful, GEIS had a limited utility to elucidate the fundamental elements that led to the high performance of the capillary-fed electrolysis cell.

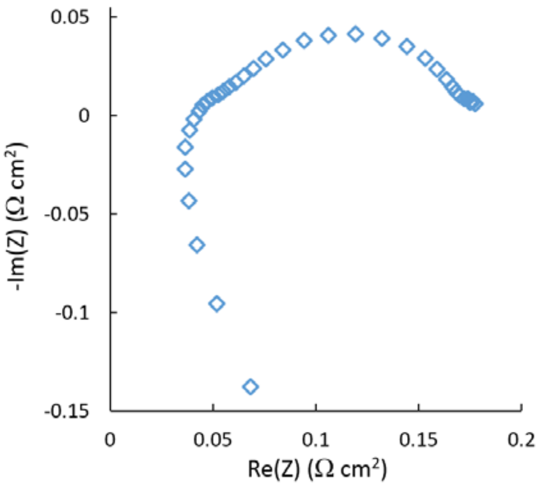

Supplementary Fig. 12

Scanning electron micrographs of the anode surface, with and without PTFE

Without PTFE

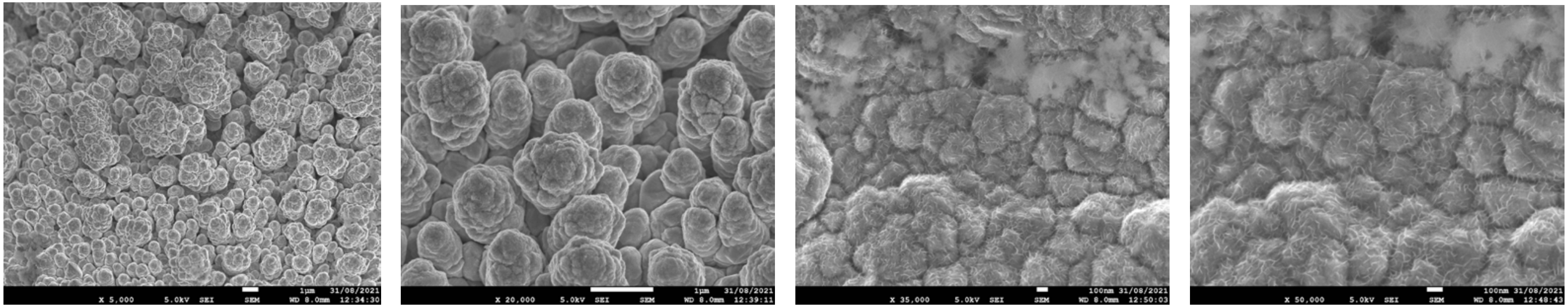

With PTFE

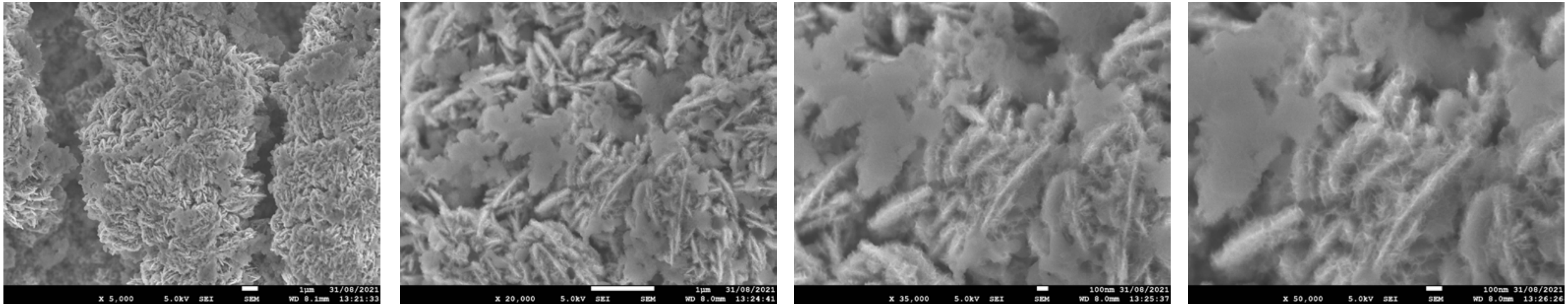

Energy-dispersive X-ray spectroscopy mapping

Without PTFE

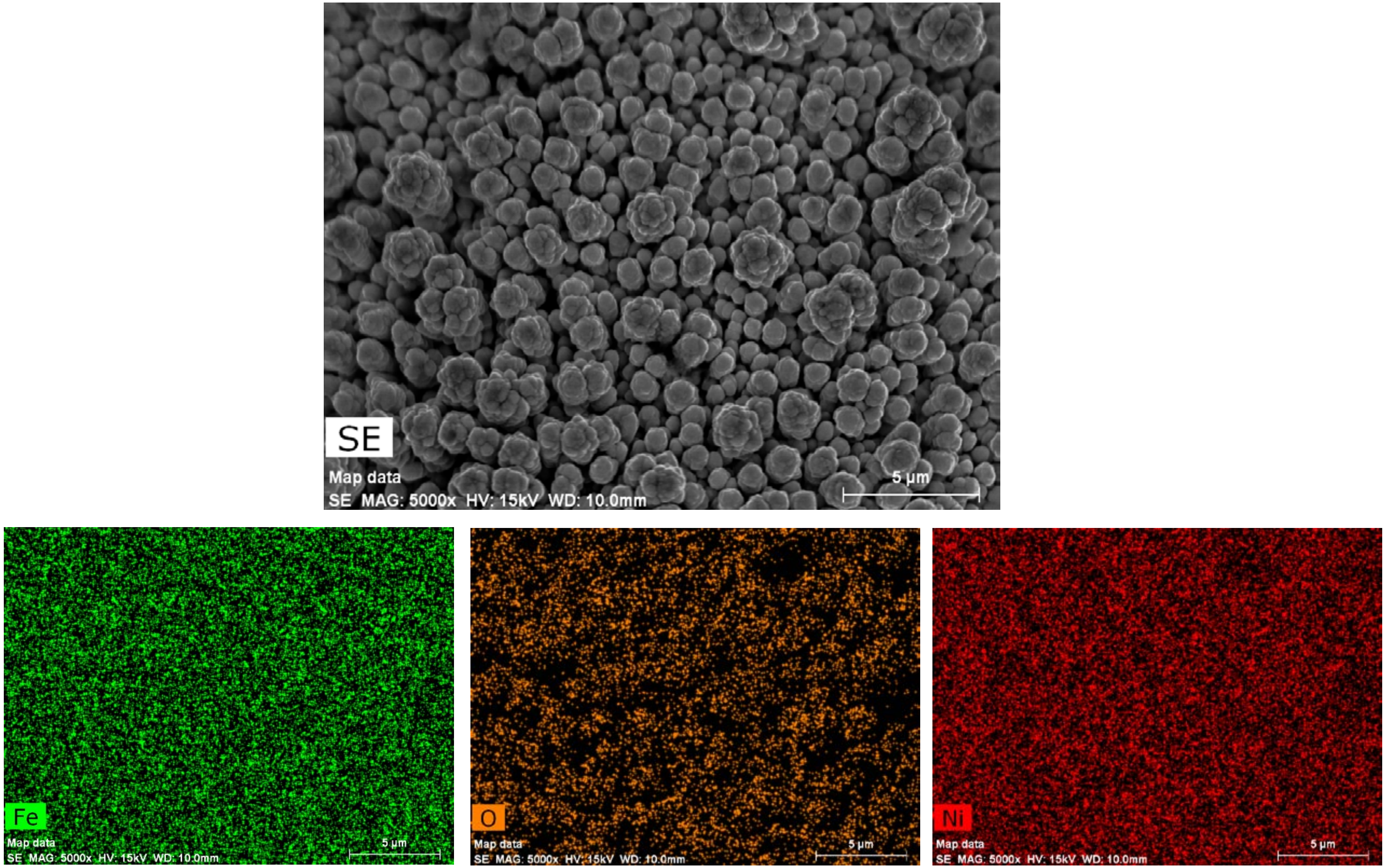

Energy-dispersive X-ray spectroscopy mapping

With PTFE

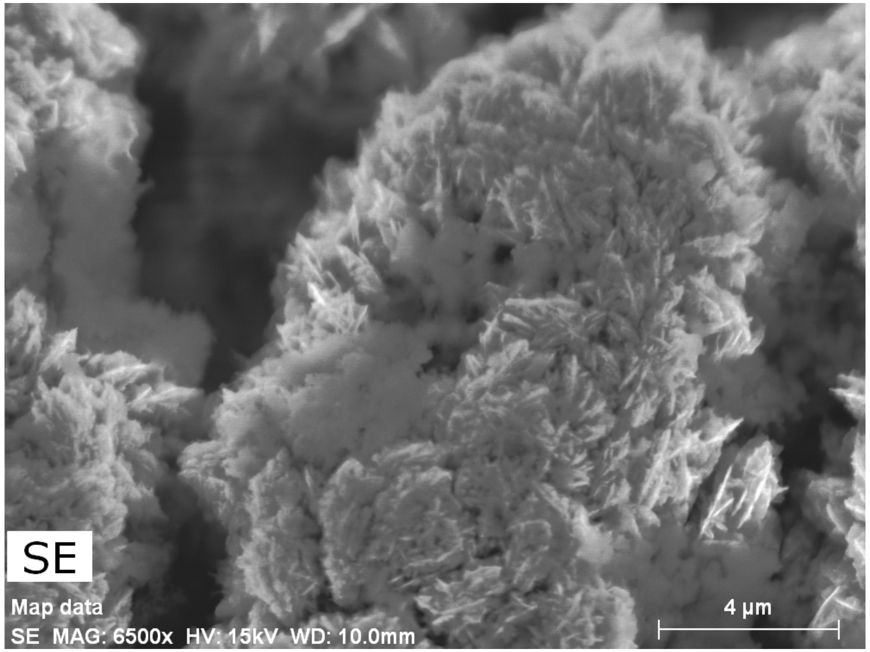

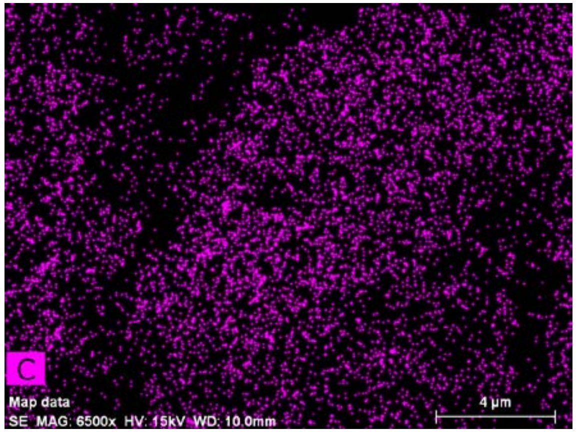

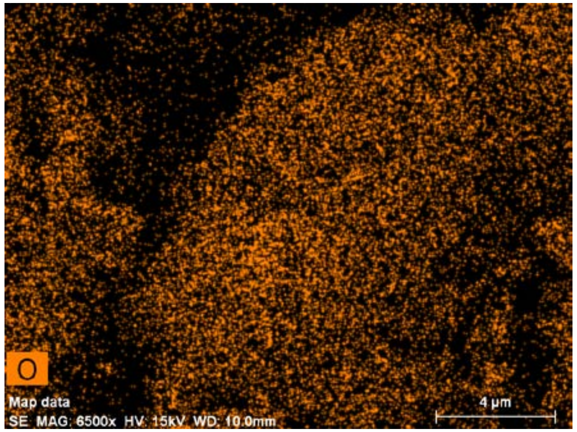

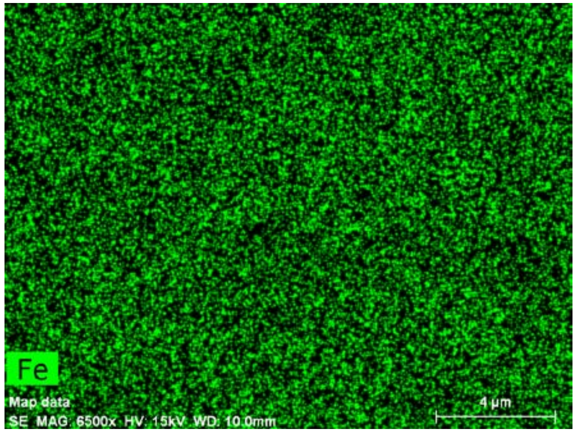

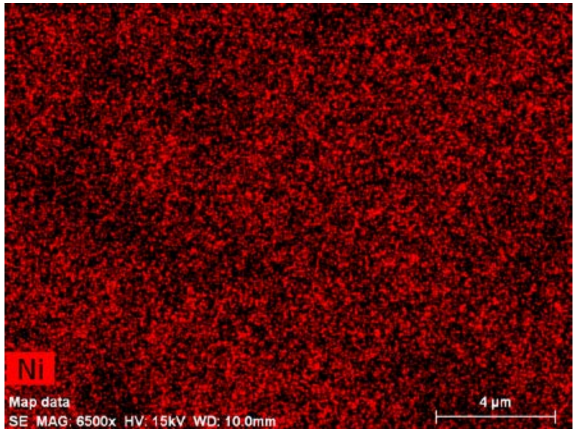

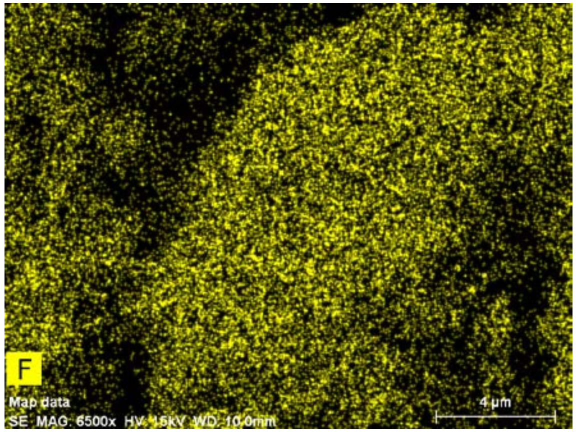

Supplementary Fig. 13

Double-layer capacitance measurements of anode with and without PTFE (in 1 M KOH)

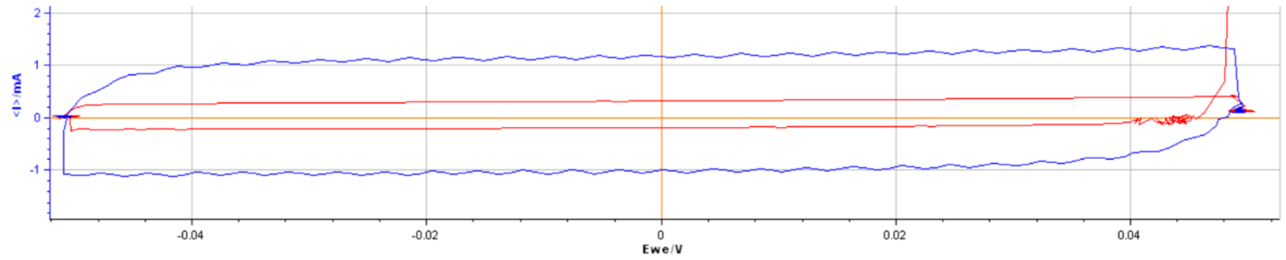

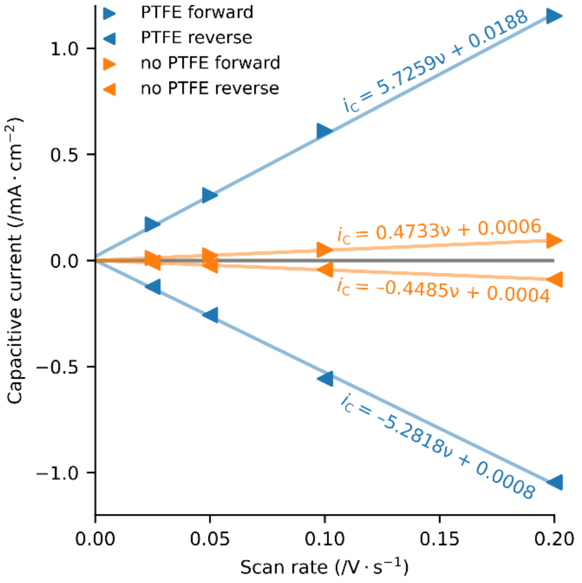

Taking the average slope of the forward and reverse sweeps, the double-layer capacitance (C_{DL}) of the anode without PTFE is calculated to be:

- 0.46 mF cm^{-2} without PTFE, and
- 5.50 mF cm^{-2} with PTFE

The double layer capacitance is therefore 5.50/0.46 = ~ 12 times larger with PTFE than without PTFE.

The electrochemically active surface area (ECSA) is related to the double-layer capacitance by the specific capacitance of the surface.

Based on the known specific capacitance of NiFe double hydroxide in 1 M KOH[14], the ECSA of the anode is ~20 cm^2 per 1 cm^2 geometric area.

As the specific capacitance of NiFe double hydroxide with incorporated PTFE is not known, the ECSA of the anode including PTFE cannot be determined.

Supplementary Table 5

Previously reported gas crossover with commercial inter-electrode separators (with equal absolute pressures on opposite sides of the separator).

Separator	Electrolyte / Liquid	Temperature (°C)	Pressure (bar)	Current density (A cm^{-2})	Concentration (H_2 in O_2) (vol.%)	Concentration (O_2 in H_2) (vol.%)	Reference
**Polyether sulfone**	**27% KOH**	**20**	**1 atm**	**0.2**	**0.06**	**0.00**	**this work**
Zirfon PERL UTP 500	30% KOH	80	10	0.4	0.21	not measured	15
Zirfon PERL UTP 500	33% KOH	40	1 atm	0.4	0.31	not measured	16
Zirfon PERL HTP 550	6 M KOH	85	30	0.1	1.20	0.30	17
Zirfon PERL HTP 550	6 M KOH	85	30	0.2	0.60	0.17	17
Zirfon PERL HTP 550	6 M KOH	85	30	0.3	0.45	0.11	17
Zirfon PERL HTP 550	6 M KOH	85	30	0.4	0.41	0.10	17
Zirfon PERL HTP 550	6 M KOH	85	30	0.5	0.41	0.11	17
Zirfon PERL HTP 550	6 M KOH	85	30	0.6	0.42	0.12	17
Zirfon PERL HTP 550	6 M KOH	85	30	0.7	0.44	0.13	17
Zirfon PERL HTP 550	6 M KOH	85	30	0.8	0.48	0.15	17
Zirfon PERL HTP 550	6 M KOH	85	30	0.9	0.55	0.16	17
Zirfon PERL HTP 550	6 M KOH	85	30	1.0	0.58	0.18	17
Zirfon PERL UTP 500	5.5 N KOH	21	1 atm	0.2	not measured	0.21	2
Asbestos (Chrysotile)	5.5 N KOH	21	1 atm	0.2	not measured	0.17	2
Nafion™ 117	DI Water	80	6	0.4	1.82	not measured	18
Nafion™ 117	DI Water	80	1 atm	0.4	1.91	not measured	19
Nafion™ 117	DI Water	80	1 atm	0.6	1.43	not measured	19
Nafion™ 117	DI Water	80	1 atm	0.8	1.10	not measured	19
Nafion™ 117	DI Water	80	1 atm	1.0	0.96	not measured	19
Nafion™ 117	DI Water	80	1 atm	1.2	0.82	not measured	20
Nafion™ 117	DI Water	80	1 atm	1.4	0.76	not measured	19
Nafion™ 117	DI Water	80	1 atm	1.6	0.70	not measured	19
Nafion™ 117	DI Water	80	1 atm	1.8	0.66	not measured	19
Nafion™ 212	DI Water	80	1 atm	2.4	1.57	not measured	19
Nafion™ 212	DI Water	80	1 atm	1.9	1.74	not measured	19
Nafion™ 212	DI Water	80	1 atm	1.4	1.91	not measured	19

Supplementary References

1. GF Swiegers, et al. Sust. Energy Fuels 5, 1280-1310
2. D Burnat, et al. J. Power Sources 291, 163-172 (DOI: /10.1016/j.jpowsour.2015.04.066)
3. RJ Gilliam, JW Graydon, DW Kirk, SJ Thorpe. Int. J. Hydrogen Energy 2007, 32, 359-64
4. Technical Data Sheet, ZIRFON PERL UTP 500, Separator membrane for alkaline electrolysis. (Agfa, 2020)
5. Porosity was measured as described in the Methods section
6. Measured using the technique in Supplementary Fig. 5
7. Imputed from the change in the specific resistance of the electrolyte
8. E Brillas, A Maestro, M Mortalla, J Casado. J. Appl. Electrochem. 1997, 27, 83-92
9. G Tsekouras, et al. Sust. Energy Fuels 2021, 5, 808-819, and references therein
10. RJ Gilliam, JW Graydon, DW Kirk, SJ Thorpe. Int. J. Hydrogen Energy 2007, 32, 359-64
11. RI Masel, Z Liu, SD Sajjid. ECS Transactions 2016, 75, 1143-1146
12. MR Kraglund, M Carmo, D Aili, G Schiller, E Christensen, A Friederich, D Stolten, JO Jensen. *Alkaline membrane electrolysis with PEM-level electrochemical performance*. Presentation at International Conference on Electrolysis 2017 (https://www.ice2017.net/conference/presentations)
13. JM Gras, P Spiteri, Int. J. Hydrogen Energy 1993, 18, 561-566
14. CL McCrory, et al., J. Am. Chem. Soc. 2013, 135, 16977-16987
15. J Otero, J Sese, I Michaus, M Santa Maria, E Guelbenzu, S Irusta, I Carrilero, M Arruebo. J. Power Sources 2014, 247, 967-974
16. IC Borbujo, et al. US patent 9,376,757
17. W Doyen, YA Gallageo, US Patent Application US2013/0337368
18. M Schalenbach, M Carmo, DL Fritz, J Mergel, D Stolten. Int. J. Hydrogen Energy 2013, 38, 14921-14933
19. M Schalenbach, G Tjarks, M Carmo, W Lueke, M Mueller, D Stolten. J. Electrochemical Society 2016, 163 (11) F3197-F3208
