# Supplementary material for: A high-performance capillary-fed electrolysis cell promises more cost-competitive renewable hydrogen
Source: Nat Commun. 2022 Mar 15;13:1304. doi: 10.1038/s41467-022-28953-x (PMC8924184; doi:10.1038/s41467-022-28953-x)
Supplement: Supplementary file 3 — Description of Additional Supplementary Files [file 41467_2022_28953_MOESM3_ESM.pdf]

Description of Additional Supplementary Files

File name: Supplementary Data 1

Description: Excel Calculator: Heat Output of Water Electrolysis Cell or Cell Stack
